# Clinical accuracy and applications of intraoral scanners in periodontal assessment: A systematic review and meta-analysis

**DOI:** 10.34172/japid.026.4282

**Published:** 2026-05-23

**Authors:** Konathala Santosh Venkata Ramesh, Saikeerthi Kalluri, Divyabhavani Vasamsetti, Sowmya Srinivas, Jagadeesan Bhuvaneswari, Nagasaireddy Konda, Guduri Vineeth, Sruthima Naga Venkata Satya Gottumukkala, Pasupuleti Swetha, Penmetsa Subhadra Gautami, Mahankali Sri Lalitha Mohana

**Affiliations:** ^1^Department of Periodontics and Implantology, Vishnu Dental College, Bhimavaram, Andhra Pradesh, India; ^2^George Mason University, Virginia, USA; ^3^Department of Public Health Dentistry, Vishnu Dental College, Bhimavaram, India; ^4^Department of Prosthodontic and Crown & Bridge, JSS Dental College and Hospital, JSS Academy of Higher Education and Research, Mysore, India; ^5^Department of Periodontics and Implantology, SreeBalaji Dental College, Chennai, India; ^6^SIBAR Institute of Dental Sciences, Guntur, Andhra Pradesh, India; ^7^Department of Prosthodontics and Crown & Bridge, Vishnu Dental College, Bhimavaram, Andhra Pradesh, India; ^8^Department of Oral and Maxillofacial Pathology, Vishnu Dental College, Bhimavaram, Andhra Pradesh, India

**Keywords:** Gingival inflammation, Gingival recession, Intraoral scanners, Periodontal diagnosis, Reproducibility

## Abstract

**Introduction:**

Intraoral scanners (IOSs) provide high-resolution 3D surface mapping and are increasingly being explored as non-invasive tools in periodontology; however, evidence on their clinical diagnostic accuracy relative to conventional standards remains variable. This study aimed to systematically evaluate the clinical accuracy, reproducibility, and applicability of IOSs in periodontal assessment.

**Methods:**

PubMed, Cochrane Library, and Google Scholar were searched until October 2025, yielding 923 records; 18 clinical studies were included following PRISMA 2020 and PROSPERO registration (CRD420251242067). Continuous plaque outcomes from three studies were pooled using inverse-variance random-effects modeling, with heterogeneity assessed using I^2^. Risk of bias ranged from low to high, with most observational studies showing moderate concerns.

**Results:**

IOS plaque scores were numerically higher than clinical references (MD=2.15; 95% CI –2.09 to 6.40), but this difference was not statistically significant (*P*=0.32; I^2^=46%). Evidence was most consistent for plaque quantification (six studies) and tissue landmark reproducibility (six studies), although certainty was low. Gingival inflammation (four studies) and gingival displacement (one RCT) showed positive trends but limited evidence. Gingival recession (three studies) and periodontal defect detection (two studies) showed very low certainty due to heterogeneity and lack of standardized validation.

**Conclusion:**

IOS enables non-invasive surface assessment and shows potential for plaque quantification and soft tissue measurements. However, evidence remains limited and heterogeneous, particularly for recession and defect detection. IOS should complement, not replace, clinical probing, and findings should be interpreted cautiously, given the limited number of quantitative studies and lack of standardization across outcomes.

## Graphical Abstract

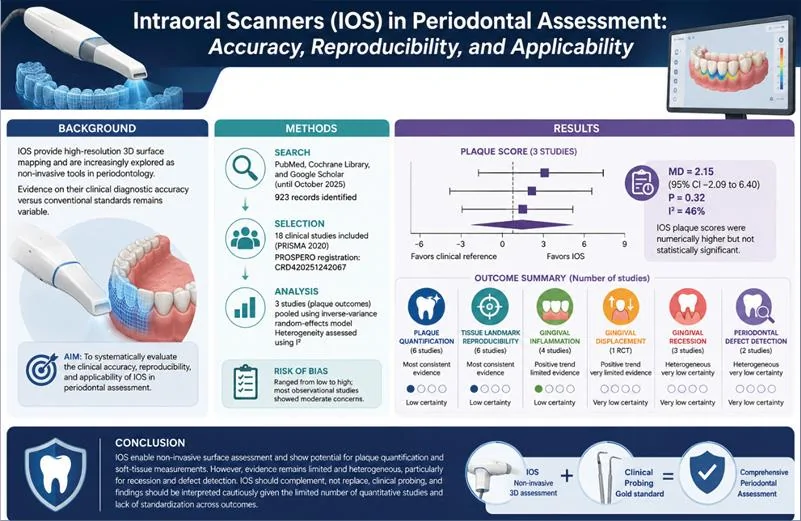

## Introduction

 The shift toward digital dentistry has steadily reshaped diagnostic workflow in periodontology, with intraoral scanners (IOSs) becoming central to this transformation. Initially designed for restorative applications, IOS technologies now offer high-resolution three-dimensional (3D) surface mapping that captures soft tissue contours, gingival recession, plaque distribution, and periodontal landmarks with increasing accuracy. Their non-invasive nature, patient comfort advantages, and ability to generate reproducible digital datasets have prompted growing interest in their potential as diagnostic tools in periodontal assessment.^1‒3^ In this context, it is important to distinguish between clinical validity, which refers to the accuracy of IOS measurements compared to established reference standards, and clinical utility, which reflects their impact on diagnostic decision-making, workflow efficiency, and patient outcomes.

 Conventional periodontal examination, relying on manual probing, radiographic evaluation, and clinician-dependent measurements, remains the reference standard to this day. However, well-documented challenges such as probe angulation errors, insertion force variability, and inter-examiner inconsistency underscore the need for adjunctive or alternative digital methods.^4‒6^ IOS-based measurements may overcome several of these limitations by providing reproducible three-dimensional datasets that enable objective quantification of tooth surfaces and soft tissue changes over time.^7‒9^ However, reported diagnostic performance varies widely depending on scanner type, software algorithms, reference standards, and clinical application.^10‒12^ In addition, IOS-based assessment has inherent limitations, particularly in detecting subgingival structures such as gingival recession depth and intrabony defects. These limitations arise from optical constraints, variability in soft tissue reflectance, and the inability to capture structures beyond the visible surface. Alternative digital modalities such as ultrasonography and transgingival probing have been explored to overcome these limitations by enabling sub-surface assessment, though they introduce their own technical and clinical constraints.

 Given this expanding but heterogeneous evidence landscape, a systematic synthesis is warranted to clarify the diagnostic accuracy and clinical applicability of IOS in periodontics. Despite heterogeneity in outcome measures across studies, certain parameters, such as plaque quantification, have been reported using comparable continuous metrics, allowing limited quantitative synthesis. Therefore, this systematic review and meta-analysis aimed to evaluate the clinical accuracy, reproducibility, and diagnostic utility of intraoral scanners, comparing them against established clinical or radiographic reference standards in periodontics.

## Methods

###  Protocol and Registration

 The review was guided by the PICO framework, where the Population (P) included adult patients or clinical periodontal sites involving natural teeth, the Intervention (I) was periodontal assessment using intraoral scanners (IOS), the Comparator (C) comprised conventional validated clinical or radiographic benchmarks such as plaque indices (stained/unstained), gingival inflammation scores, gingival recession or attachment measurements, periodontal phenotype analysis, tooth mobility evaluation, and periodontal defect or bone-level assessments performed clinically, photographically, or via cone-beam computed tomography (CBCT), and the Outcome (O) was the quantitative accuracy, reliability, reproducibility, or clinical applicability of IOS-derived periodontal measurements and diagnostics; based on this, the protocol was developed according to PRISMA 2020 guidelines and prospectively registered in PROSPERO (https://www.crd.york.ac.uk/PROSPERO/view/CRD420251242067) to ensure methodological transparency and reproducibility, with the primary research question defined as: What is the clinical quantitative accuracy, reliability, and applicability of intraoral scanners in periodontal assessment and diagnosis compared with conventional reference standards?

###  Eligibility Criteria

 Eligibility criteria included human clinical studies that assessed intraoral scanners for periodontal applications, with evaluation of at least one of the following parameters: gingival recession, clinical attachment loss, plaque, tooth mobility, periodontal defects, gingival morphology, or alveolar process visualization. Comparative or validation studies using clinical or radiographic gold standards (such as periodontal probing, caliper measurements, or CBCT) were included. Studies were restricted to English-language publications with full-text availability. Exclusion criteria comprised animal or in vitro studies, studies focusing solely on restorative or prosthodontic accuracy, orthodontic scanning, ultrasound imaging, implant scan bodies, as well as case reports, narrative reviews, editorials, and conference abstracts. Studies involving artificial intelligence-based periodontal assessment were included only if their outputs were validated against established clinical or radiographic reference standards.

###  Information Sources and Search Strategy

 Literature searches were performed in PubMed, the Cochrane Library, and Google Scholar from database inception until October 2025. Search strategies and corresponding yields are listed in Table 1. Manual reference list screening was additionally performed to capture relevant periodontal IOS studies not retrieved through database queries. Google Scholar was used as a supplementary source to capture grey literature. All retrieved records (n = 662) were systematically screened by title and abstract. Non-relevant, duplicate, and non-peer-reviewed sources were excluded based on predefined eligibility criteria.

**Table 1:** Database search strategy and screening yield

**Database**	**Search terms used**	**Records Retrieved**
PubMed	("Intraoral Scanners"[Mesh] OR "Intraoral Scanner*" OR "Digital Impression*" OR "Optical Impression*" OR "3D Scan*" OR "IOS") AND (“Periodontal Diseases” [Mesh] OR “Periodontal Assessment” OR “Gingival Recession” OR “Clinical Attachment Loss” OR “Plaque” OR “Tooth Mobility” OR “Periodontal Defect*” OR “Gingiva” OR “Alveolar Process”) AND (human [Mesh] AND English [lang])	161
Cochrane Library	("intraoral scanner*" OR "digital impression*" OR "optical dental scan*") AND (“periodontal” OR “gingival recession” OR “attachment loss” OR “dental plaque” OR “tooth mobility” OR “periodontal defect*” OR “alveolar bone” OR “gingiva”)	100
Google Scholar	(“intraoral scanner” OR “digital impression” OR “3D dental scan”) AND (“periodontal” OR “gingival recession” OR “attachment loss” OR “tooth mobility” OR “periodontal defect*” OR “plaque” OR “alveolar bone”) AND (“accuracy” OR “diagnostic” OR “measurement” OR “reliability” OR “validity”) NOT “prosthodontics” NOT “orthodontics” NOT “crown” NOT “implant scan body”	662

###  Study Selection

 All retrieved records were imported into EndNote X9 for deduplication, and two independent reviewers screened the titles and abstracts for relevance. Full texts of potentially eligible studies were retrieved and assessed against the inclusion and exclusion criteria. Disagreements between the two independent reviewers were resolved through discussion to reach consensus or, if needed, through consultation with a third reviewer. Inter-reviewer agreement was assessed using Cohen’s kappa coefficient, demonstrating strong agreement. Reviewers were calibrated before screening to ensure consistency. For the two studies that could not be retrieved, attempts were made through institutional access and by contacting corresponding authors.

###  Data Extraction

 Data extraction was performed using a standardized form that included information on the author and year of publication, study design, sample size, population characteristics, type and model of intraoral scanner, periodontal parameters assessed, comparator methods, outcomes (accuracy, reproducibility, mean deviations), and key findings. Data extraction was conducted independently by two calibrated reviewers to ensure consistency and minimize bias, with discrepancies resolved through consensus. Extracted variables included scanner-related error metrics, reference standards used, and quantitative deviation outcomes.

###  Quality Assessment

 Risk-of-bias assessments for the included studies, including traffic-light plots and summary graphs, were generated using the robvis (Risk-of-bias Visualization) tool. For consistency, domain-level judgments were harmonized across studies using the QUADAS-2 domains. Domain-level judgments were summarized and displayed in a single traffic-light evidence plot, while overall evidence certainty was interpreted using the GRADE framework to guide confidence in the clinical applicability and the synthesized periodontal relevance of intraoral scanner technologies for periodontal assessment and diagnosis.

###  Statistical Analysis

 For outcomes reporting continuous quantitative data, a meta-analysis was conducted using mean differences (MD) with 95% confidence intervals via inverse-variance random-effects modeling in Review Manager (RevMan) software (version 5.4, The Cochrane Collaboration, Copenhagen), and the results were presented as forest plots. Overall pooled significance was interpreted using Z-tests with *P* < 0.05, and heterogeneity was quantified using I^2^ statistics.

 Given the heterogeneity in outcome definitions and measurement scales, meta-analysis was restricted to plaque-related outcomes reported as continuous variables. Due to the limited number of studies, subgroup and sensitivity analyses were not feasible, and publication bias was not assessed. Therefore, findings from the quantitative synthesis should be interpreted with caution.

## Results

 A total of 923 articles were retrieved across search databases (PubMed, Cochrane Library, and Google Scholar). After removing 16 duplicates using EndNote X9, 907 records underwent title and abstract screening, resulting in the exclusion of 864 non-relevant articles. Forty-three articles were assessed for full-text eligibility, 2 of which could not be retrieved. Thirty-nine full texts were evaluated, 21 of which were excluded based on eligibility criteria. Ultimately, 18 studies that fulfilled the inclusion criteria and addressed the primary research question were selected for qualitative synthesis (Figure 1).

**Figure 1:**
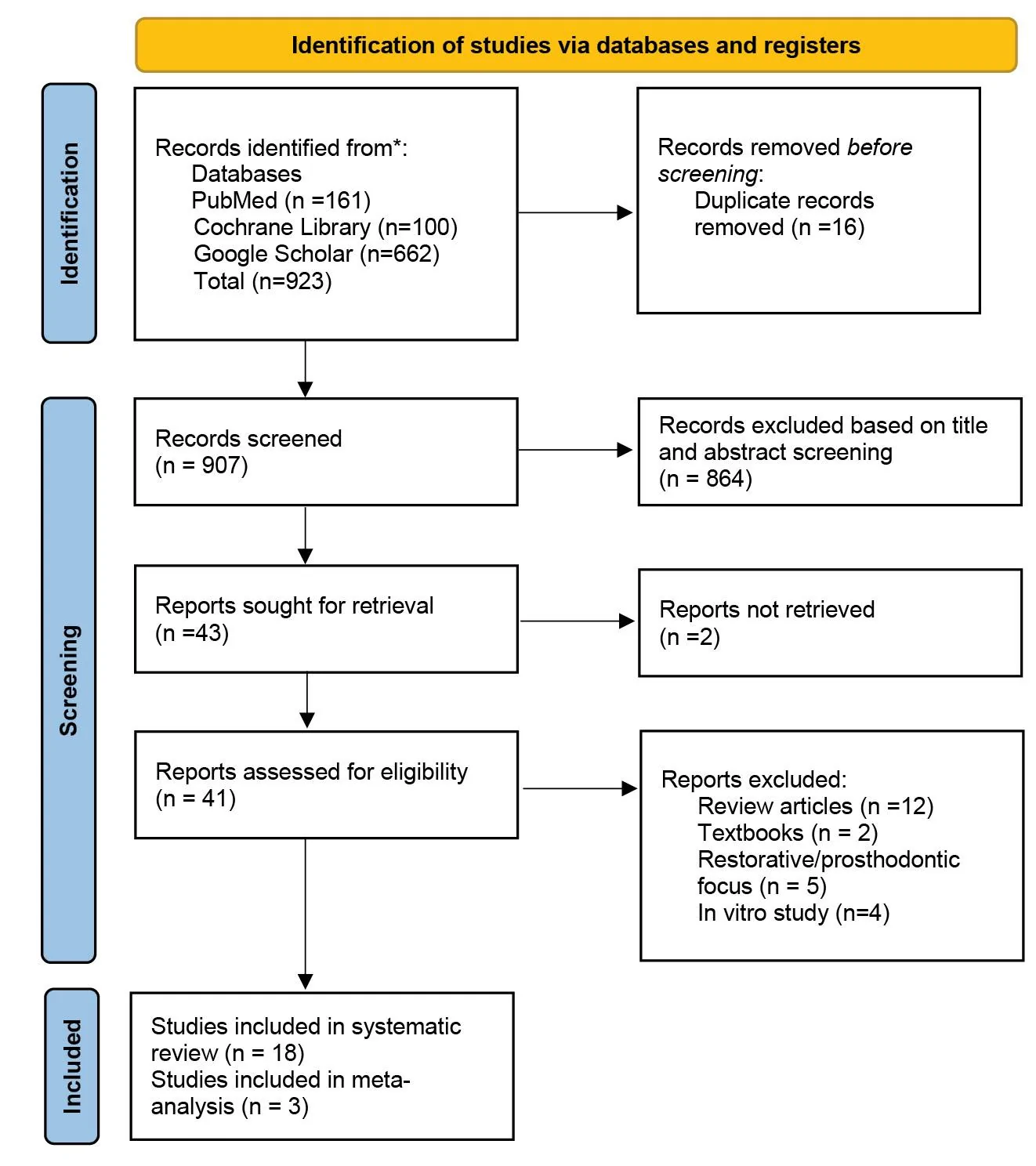
PRISMA flowchart of included studies

###  Characteristics of Included Studies

 The current review included 18 human clinical studies published in English up to 2025, which assessed intraoral scanners for periodontal measurement and diagnosis. Most studies had analytical observational designs, while 4 incorporated machine-learning or digital fusion workflows for periodontal diagnosis. IOS was primarily applied for dental plaque visualization, gingival contour evaluation, periodontal phenotype measurement, gingival inflammation analysis, and periodontal defect or gingiva-bone distance assessment. Study samples ranged from small single-center clinical cohorts (n = 20‒120) to large multicenter digital datasets (n > 2,500). Large datasets (n > 2,500) represented clinical datasets used for model development and validation rather than purely retrospective archival data (Table 2).^13‒30^

**Table 2:** Characteristics of included periodontal intraoral scanner (IOS) studies

**Author**	**Study Design**	**Sample Size & Population**	**Intraoral Scanner (Model/** **Generation)**	**Periodontal Parameters Assessed**	**Reference Standard Used**	**Primary Outcomes (Accuracy / Deviations)**	**Key Findings / Clinical Applicability**
Newby et al.^13^	Randomized controlled trial	Adult participants receiving randomized quadrant dental prophylaxis; follow-up at 1, 2, 4, 6, 12 weeks	LAVA^TM^ Chairside Oral Scanner (COS)	Gingival contour and volume changes from digital impressions (quantitative 3D gingival morphology)	Traditional periodontal indices: Modified Gingival Index (MGI) and Bleeding Index (BI); also, oral microflora analysis	Gingival contour/volume reductions in prophylaxis quadrants matched significant improvements in MGI and BI up to 6 weeks. Digital impressions could detect gingival volume changes parallel to clinical inflammatory reduction.	Digital gingival volume measurement is a novel, objective, and sensitive method for assessing gingival health. IOS-based volumetric analysis correlates with traditional indices and can quantify gingivitis improvement after prophylaxis.
Ioshida et al.^14^	Analytical study	12 adults undergoing orthodontic treatment (pre- & post-treatment datasets)	Intraoral scanner generating Digital Models (DM) (model not specified)	Mucogingival junction used as registration landmark; mandibular model positional changes (RL, AP, SI, 3D)	High-resolution CBCT voxel-based registration (gold standard)	No significant differences between CBCT and DM registrations (*P* = 0.076–0.384); excellent reproducibility (ICC > 0.90)	Mandibular digital model registration using the mucogingival junction is accurate, reliable, and reproducible, providing a valid method for digital model alignment in orthodontics/periodontics.
Wei et al.^15^	In vivo study	5 participants with full upper dentition	3Shape TRIOS Color (TRC); CEREC Omnicam (OC)	Gingival contour accuracy in the esthetic maxillary zone	Conventional impressions (vinyl polysiloxane) digitized using a model scanner (IScanD103i)	Precision: TRC 45.10 ± 12.54 µm; OC 66.04 ± 13.46 µm; CI 63.66 ± 17.19 µm. Trueness: TRC 80.12 ± 8.69 µm; OC 82.70 ± 8.85 µm	Both IOS systems showed clinically acceptable accuracy for gingival contour capture. TRC demonstrated higher precision than OC and CI. IOS is reliable for esthetic zone soft-tissue documentation.
Daly et al.^16^	observational study	23 adults (552 gingival sites) with a spectrum of gingival inflammation	Intraoral scanner (model not specified)	Modified Gingival Index (MGI) scoring from IOS images	Chairside clinical MGI scores	90% agreement between clinical vs IOS-derived MGI; Most common errors: clinical 0 → IOS 1 and clinical 2 → IOS 3; IOS MGI between two examiners showed 91% agreement, no discrepancies > 1 grade	IOS accurately captures gingival contour and inflammation features sufficient for MGI scoring; a valid non-invasive method for remote assessment, research use, and digital monitoring of gingival inflammation.
Giese-Kraft et al.^17^	In vivo study	20 adults (mean age 27.5 ± 1.2 years)	Carestream CS 3600 (3D IOS) and CS 1500 intraoral camera (2D)	Dental plaque levels on RMNPI index areas at T1, T2, T3	Clinical plaque examination using Rustogi-modified Navy Plaque Index (RMNPI)	Mean plaque % T1: Clinical 62.2%, 2D 65.1%, 3D 64.4; T2: Clinical 76.9%, 2D 77.9%, 3D 77.5; T3: Clinical 56.3%, 2D 58.2%, 3D 61.2. Good agreement in Bland-Altman; substantial–almost perfect agreement per RMNPI area	2D (intraoral camera) and 3D (IOS) imaging reliably detect and monitor disclosed plaque. Digital imaging shows high agreement with clinical examination and enables objective longitudinal plaque monitoring.
Jung et al.^18^	Analytical study	N = 20 adults (mean age 27.5 ± 1.2 years); plaque assessed at T1 habitual, T2 72h no hygiene, T3 post-brushing	CS 3600 intraoral scanner (Carestream Dental, Germany)	Dental plaque planimetry (% plaque-covered surface area) on oral & vestibular surfaces of Ramfjord teeth (16, 21, 24, 36, 41, 44)	Intraoral camera CS 1500 (Carestream) for vestibular surfaces of teeth 16 and 36	Strong correlation between IOS images and reference camera: r = 0.876; *P* < 0.001. Bland–Altman: good agreement, minor systematic bias. P% increased 47% from T1→T2; decreased 43% after brushing (T3) (*P* < 0.001).	3D IOS images valid for whole-mouth plaque planimetry; accurate, reliable, sensitive to plaque changes; useful for monitoring oral hygiene and research applications.
Gunpınar et al.^19^	mixed-method study	1057 volunteers—demographic + full clinical periodontal dataset; intraoral scans used for individualized simulation	Intraoral scanner (model not specified) used to generate direct optical 3D intraoral models	Periodontal risk classification (based on clinical parameters), gingival recession simulation (gingival margin modeling)	No traditional “gold standard”; clustering (K-means) used to classify periodontal risk; clinical exam used as baseline dataset	The algorithm successfully extracted gingival–tooth separation curves using Dijkstra’s algorithm. Software-generated patient-specific recession models based on user-defined recession input. Population stratified into low (n = 462), medium (n = 336), and high (n = 259) periodontal-risk groups.	A clinically usable digital periodontal risk prediction and gingival recession simulation tool was developed. Enables clinicians to visually demonstrate individualized recession progression, improving patient motivation, understanding, and preventive behavior.
Daly et al.^20^	Randomized Controlled Trial (RCT)	Adult participants with pre-existing gingivitis (number not explicitly stated in abstract)	Intraoral Scanner (IOS) – model not specified	Bleeding on Probing (BOP), Plaque Scores	Clinical periodontal exam; IOS images for visualization	Significant reduction in BOP and plaque scores in the intervention group compared to control at all visits (*P* < 0.001 for BOP; *P* < 0.05 for plaque)	IOS-guided oral hygiene advice + anti-gingivitis toothpaste + motivational reminders significantly improved gingival health more than standard care. Supports the use of IOS for behavior change and monitoring in clinical practice.
Karaduman et al.^21^	Analytical study	32 individuals; maxillary anterior region (canine to canine)	Digital scanner + 3D measuring tool (specific model not reported)	Gingival Zenith–Vertical Midline distance (GZVM) & Gingival Zenith of lateral incisor (GZLI)	Analog plaster casts measured with a digital caliper	ICC for GZVM: 0.366–0.755 (moderate to good agreement). ICC for GZLI: 0.788 (right), 0.395 (left).	Digital and analog methods showed compatible measurement accuracy for gingival zenith positions; either method is acceptable depending on equipment, time, and operator preference.
Guo et al.^22^	Non-Randomized Controlled Trial	140 teeth from 5 healthy subjects with complete dentition	IOS (exact model not reported) was used to obtain color 3D images	Dental plaque staining area; Quigley-Hein Plaque Index (PI) at T1 (no cleaning 24 hrs) & T2 (post-brushing)	Clinical Plaque Index (PI) scoring by the examiner	Strong correlation with clinical PI: Spearman r = 0.9136 (T1), 0.9061 (T2); Excellent inter-examiner agreement: ICC = 0.989–0.992 (T1), 0.964–0.983 (T2)	Digital 3D plaque analysis using IOS is highly accurate, reliable, and reproducible; Suitable for clinical practice & research, enabling quantitative plaque assessment
Doi et al.^23^	single-arm clinical trial	20 patients with > 20% plaque control record (PCR)	Intraoral scanner (model not specified; color IOS)	O’Leary’s Plaque Control Record (PCR) across full dentition and sextant-specific regions	Direct clinical visualization of plaque after disclosing gel	IOS-based PCR values tended to be higher than the direct visual method; no significant difference for: maxillary anterior labial/palatal surfaces and mandibular anterior labial/lingual surfaces	IOS enables clear visualization of disclosed plaque, promising for plaque documentation and hygiene monitoring. Large tip size limits access; smaller IOS tips are needed for improved accuracy in confined areas.
Sun et al.^24^	Analytical study	10 healthy volunteers; palatal mucosa of maxillary anterior teeth	IOS (model not specified) combined with CBCT	Palatal mucosal thickness at multiple gingival reference points	CBCT-based indirect gingival imaging & modified soft tissue CBCT (ST-CBCT)	Mean differences between methods < 0.2 mm; < 5% points outside 95% LoA; ICC > 0.75for all methods (high reproducibility)	CBCT + IOS provides clinically reliable and reproducible palatal mucosal thickness measurements; feasible for donor area evaluation in soft tissue augmentation.
Gottumukkala et al.^25^	Cross-sectional study	30 periodontally healthy adults (20–25 yrs)	IOS used to generate STL files (model not specified), combined with CBCT DICOM data	Gingival thickness (GT) and bone thickness (BT) at 2 mm & 4 mm apical to the bone crest	Direct Technique (DT): Transgingival probing with endodontic file + digital caliper	Very high correlation between digital vs DT measurements (r = 0.86 for tooth-wise; r > 0.8 for all GT & BT measures)	Digital analysis using STL–DICOM superimposition is reliable, non-invasive, and comparable to the direct transgingival probing technique for periodontal phenotype assessment.
Tan et al.^26^	Multicenter diagnostic system development and validation study	2,507 patients across multicenter cohorts; IOS + CBCT datasets used to develop and validate PerioAI	Intraoral scanner used for IOS segmentation (specific model not stated; integrated into PerioAI workflow)	Gingiva-Bone Distance (GBD) measurement; digital periodontal probing; soft + hard tissue segmentation and fusion	CBCT imaging used as the anatomical reference for hard tissue; integrated multimodal fusion with IOS	PerioAI achieved 0.040 mm mean error for digital probing measurements. Outstanding IOS + CBCT segmentation accuracy contributed to reliable full-stack periodontal analysis.	PerioAI provides an accurate, automatic, non-invasive periodontal diagnostic system. Enables digital probing of GBD with extremely high precision. Offers potential to enhance periodontal diagnosis, reduce clinician burden, and improve treatment planning accuracy.
Li et al.^27^	Analytical study	120 patients with periodontitis; IOS images + periodontal probing data	Intraoral scanner used for IOS imaging (model not specified)	Gingival inflammation surface features: correlations with SBI, BI, PD	Clinical periodontal probing indices (SBI, BI, PD) as reference clinical indicators	GC-U-Net segmentation performance: Dice 77.8%, IoU 65.4%, Pixel Accuracy 93.7%; Strong correlation with SBI (r = 0.836), moderate with BI (r = 0.618), negative with PD (r = –0.425)	Deep-learning + IOS system accurately identifies gingival inflammation regions. Provides objective, standardized assessment, reducing subjective bias and supporting clinical diagnosis and monitoring of gingival inflammation.
Meseli et al.^28^	cross-sectional study	28 volunteers, divided into low DMFT ( < 3) and high DMFT ( > 10) groups	Intraoral digital scanner (specific model not stated)	Dental plaque scores on anterior labial surfaces using Turesky Modified Quigley–Hein Plaque Index	Clinical visual examination (with and without plaque staining)	In the low DMFT group, stained clinical exam scores were similar to digital photography (*P* = 1.000) and intraoral scanner scores (*P* = 0.997). FluoreCam produced the highest plaque scores across both groups. Visual exam without staining produced lowest scores (*P* < 0.05).	Plaque staining increases diagnostic efficiency in low DMFT patients. Intraoral scanners can serve as an alternative plaque-scoring tool, comparable to stained clinical exams; FluoreCam detects more plaque quantitatively.
El Ashry et al.^29^	Randomized Controlled Trial	32 participants with maxillary premolars requiring full-coverage crowns	Digital intraoral scanner (brand/model not specified)	Horizontal gingival displacement Vertical gingival displacement Sulcus depth Gingival height loss (7, 15, 30 days post-cementation)	Baseline pre-operative digital impression used as reference for displacement & height change	Impregnated cord (RCA) showed highest displacement: 0.66 ± 0.04 mm (horizontal), 0.66 ± 0.008 mm (vertical). Other methods (Magic FoamCord, Expasyl, Laser) ranged 0.24–0.48 mm. RCA caused the highest gingival height loss after 1 month (*P* < 0.001).	Impregnated cords provide superior gingival displacement for digital impressions but cause greater gingival height loss. Magic Foam Cord offers a safer compromise. Expasyl and laser produce less displacement. For digital impressions of subgingival margins, cords or non-astringent paste with compression caps are effective but must avoid trauma.
Thießen et al.^30^	Analytical study	N = 20 subjects; adults aged 18–58 years; maxillary and mandibular double scans taken consecutively	Intraoral scanner(model not specified; used for digital maxilla and mandible scans)	Hard- and soft-tissue linear measurements between predefined anatomical points	None (reproducibility assessed by comparing 1st vs 2nd digital scan using 3 superimposition methods)	No significant differences in linear distances between first and second scans for both hard & soft tissues (paired t-test, *P* < 0.05). No significant differences between 3 matching/superimposition methods (soft tissue *P* = 0.196; hard tissue *P* = 0.963).	Digital intraoral scans provide reproducible measurements of hard and soft tissues. All 3 scan-matching methods are suitable for superimposition. Reliability depends on operator skill, system limitations, and patient factors.

###  Quality Assessment 

 The overall risk-of-bias assessment indicated some concerns, largely due to inconsistencies in reference standard validation and incomplete reporting of flow and follow-up in several observational studies (Figures 2 and 3). Most RCTs showed low risk across domains, except for Gunpınar et al.^19^ (2025) and Tan et al.^26^ (2025), both of which were rated high risk due to issues with patient selection, index test conduct, and applicability of the reference standard. High risk of bias in certain studies was primarily attributed to lack of blinding, variability in segmentation algorithms across software versions, and operator-dependent measurement variability.

**Figure 2:**
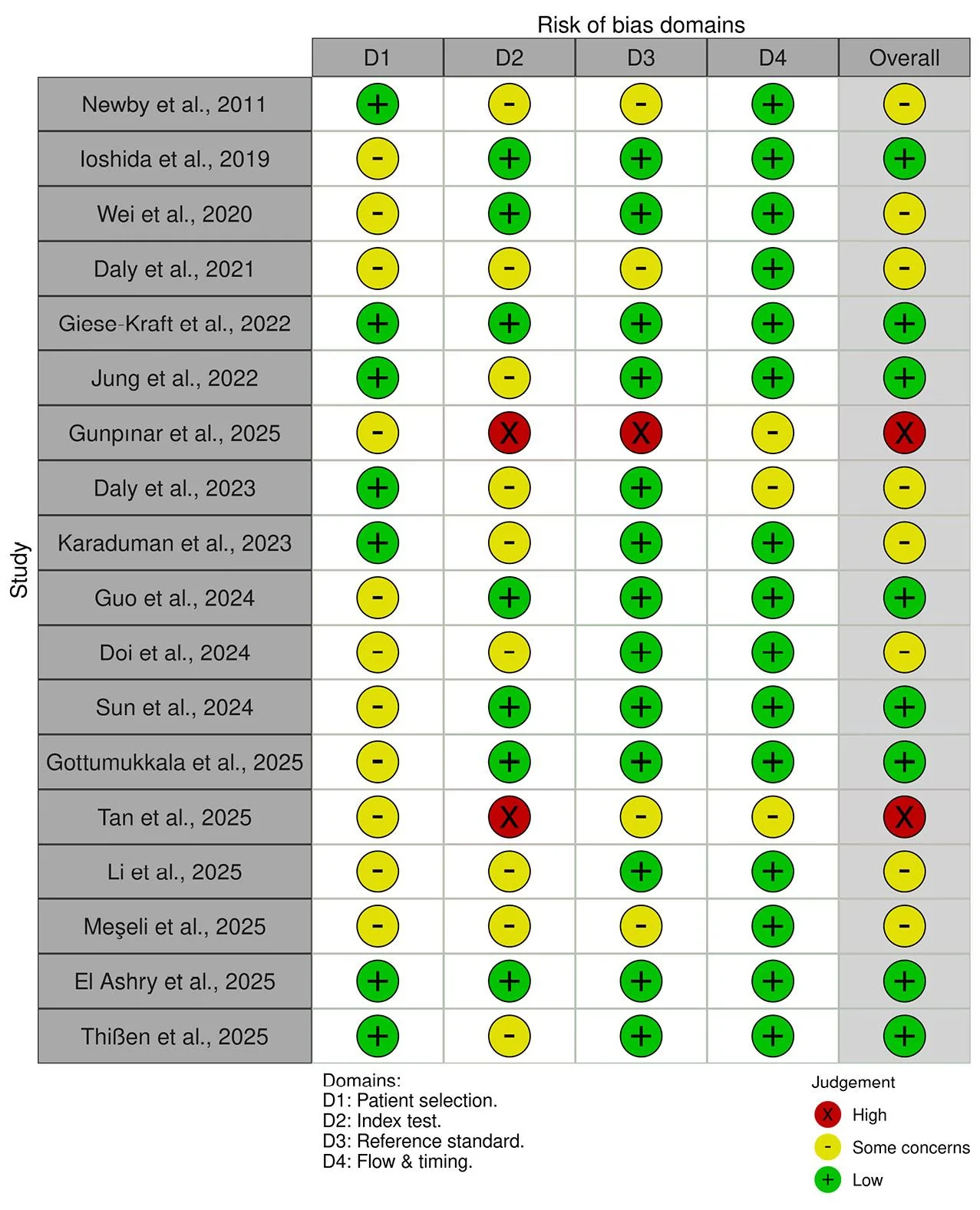
Risk of bias summary (Newby et al.^13^, Ioshida et al.^14^, Wei et al.^15^, Daly et al.^16^, Giese-Kraft et al.^17^, Jung et al.^18^, Gunpınar et al.^19^, Daly et al.^20^, Karaduman et al.^21^, Guo et al.^22^, Doi et al.^23^, Sun et al.^24^, Gottumukkala et al.^25^, Tan et al.^26^, Li et al.^27^, Meseli et al.^28^, El Ashry et al.^29^, Thießen et al.^30^)

**Figure 3:**
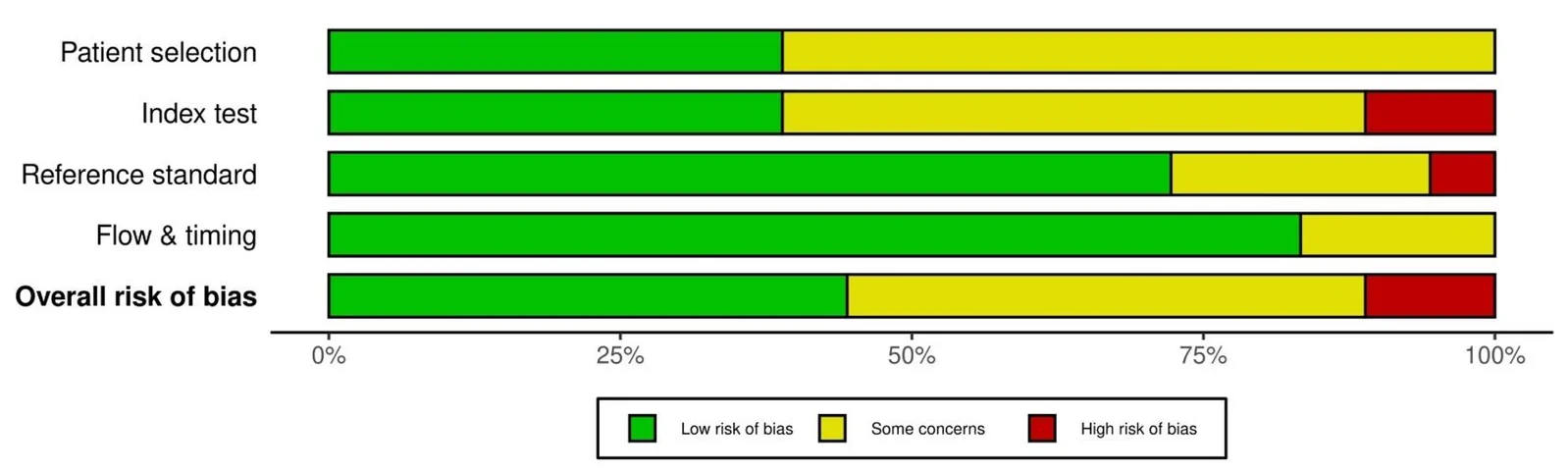
Risk of bias graph

 Across the observational evidence base, moderate concerns were common, particularly in the reference standard domain, where several studies used less robust or variably validated comparators. Reproducibility-focused studies (e.g., Ioshida [2019] and Newby [2011]) demonstrated a low overall risk, though limitations of the reference standard remained the most frequent source of bias. Overall, the traffic-light plots indicate that the primary drivers of bias stem from weaknesses in the reference standard, sampling uncertainty, and variability in follow-up reporting, rather than inconsistencies in diagnostic performance.

###  Evidence Grading

 Certainty of evidence was assessed using the GRADE approach. Eighteen included human studies evaluated intraoral scanners (IOS) for periodontal use. IOS demonstrated relatively consistent evidence for plaque quantification accuracy (six studies) and reproducible soft and hard tissue measurements at periodontal landmarks (six studies); however, the certainty of this evidence was low due to imprecision and heterogeneity across studies. Gingival inflammation assessment (four studies) and sulcus/crown margin displacement effects (1 small RCT, n = 32) showed positive trends but were based on small, largely unblinded clinical samples, resulting in low to very low certainty. Gingival recession accuracy (three studies) and periodontal defect or gingiva-bone/CBCT-AI IOS fusion approaches (two studies) lacked uniform, direct in vivo validation against routine periodontal probing and were therefore associated with very low certainty. IOS is currently the most reliable tool for non-invasive quantitative measurements, while other diagnostic applications remain preliminary and should be interpreted with caution (Table 3).

**Table 3:** Quality appraisal of included studies with GRADE analysis

**Outcome**	**No of studies **	**Risk of bias**	**Inconsistency**	**Indirectness**	**Imprecision**	**Other considerations**	**No of patients (IOS/** **reference)**	**Certainty (GRADE)**
Plaque quantification accuracy of IOS vs clinical reference	5 studies (Jung et al.^18^, Giese-Kraft et al.^17^, Guo et al.^22^, Doi et al.^23^, Meşeli et al.^28^)	Not serious	Serious	Not serious	Serious	None	150–180 with IOS and clinical reference	⨁⨁◯◯ **Low**
Gingival recession digital measurement accuracy	Gunpınar et al.^19^	Serious	Not estimable	Serious	Serious	None	25–50 in clinical volumetric studies; 1057 in simulation dataset	⨁◯◯◯ Very Low
Gingival inflammation / gingival health diagnostic accuracy from IOS images	4 studies (Newby et al.^13^, Daly et al.^16^, Daly et al.^20^, Li et al.^27^)	Serious	Serious	Not serious	Serious	None	250–300	⨁◯◯◯ Very Low
Effect of crown margin / gingival displacement for IOS on short-term recession	El Ashry et al.^29^	Some concerns	Not applicable	Not serious	Serious	None	32 (all received IOS impressions after different displacement methods)	⨁⨁◯◯ Low
Periodontal defect / bone level detection accuracy (IOS ± CBCT)	Tan et al.^26^	Serious to very serious	Not assessable	Serious	Serious	None	2507 clinical patients in PerioAI; in-vitro sample around extracted teeth	⨁◯◯◯ Very Low
Soft and hard tissue measurement reproducibility (periodontal phenotype, mucosa thickness, gingival zenith, model registration)	6 human studies – Thißen et al.^30^, Guo et al.^22^, Gottumukkala et al.^25^, Ioshida et al.^14^, Sun et al.^24^, Karaduman et al.^21^	Not serious	Not serious	Not serious	Not serious	None	120–150	⨁⨁◯◯ **Low**

###  Meta-analysis on Plaque Score Assessment Using IOS Versus Clinical Reference

 Three disclosed-plaque imaging studies contributed quantitative data for meta-analysis.^17,23,28^ At baseline and endpoint, the overall mean plaque surface scores were numerically higher in the IOS group (MD = 2.15; 95% CI: –2.09 to 6.40); however, the pooled difference was not statistically significant (Z = 0.99, *P* = 0.32). The overall heterogeneity from baseline to endpoint was moderate (I^2^ = 46%), and a random-effects model was applied due to variations in disclosure methods, scanned regions, and digital analysis workflows (Figure 4). The remaining 15 studies were not pooled because they used ordinal severity indices, segmentation outputs, or simulation/AI fusion models without uniform continuous deviation metrics. The clinical relevance of the observed mean difference remains uncertain due to the lack of standardized units across studies. Although standardized mean differences (SMD) are generally recommended when outcome scales differ, the included studies assessed conceptually similar plaque indices; therefore, mean difference (MD) was retained to preserve clinical interpretability. Given that only three studies were included in the quantitative synthesis, the statistical power of the meta-analysis is limited.

**Figure 4:**
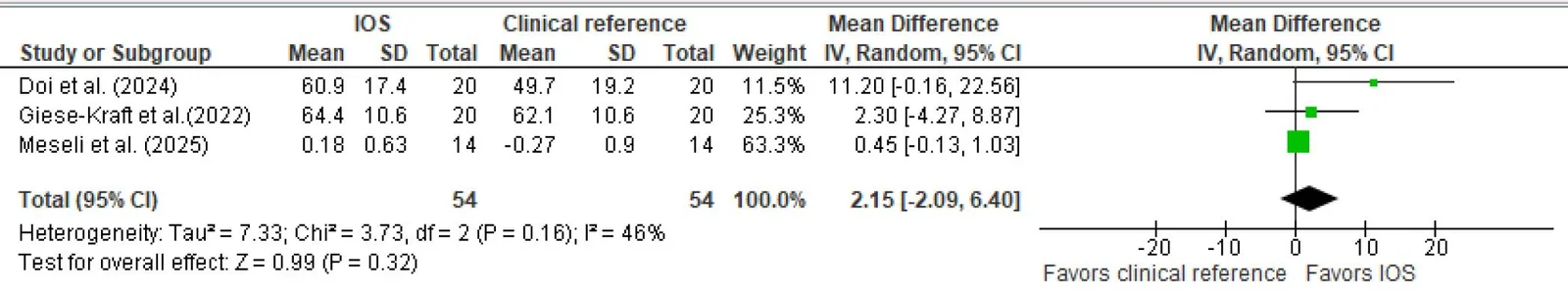
Meta-analysis of plaque score assessment using IOS compared with clinical reference (Doi et al.^23^ (2024), Giese-Kraft et al.^17^ (2022), Meseli et al.^28^ (2025)).

## Discussion

 Comprehensive diagnostic records are essential for treatment planning, evaluation, and legal documentation.^31^ Digital models provide a cost-effective alternative to conventional casts, enabling assessment of tooth movement and periodontal biotype, as well as virtual simulations, with seamless integration into CBCT and other digital records.^14^ However, as soft tissues cannot be accurately replicated in vitro, in vivo validation of intraoral digital impressions is necessary.^1,15,32,33^

 Periodontitis remains highly prevalent worldwide, with delayed diagnosis largely due to reliance on manual periodontal probing, which is subjective, technique-sensitive, and time-consuming.^34^ While radiographs aid in assessing alveolar bone loss, the need to integrate clinical and radiographic findings increases diagnostic complexity.^26^ Advances in intraoral scanning (IOS) now enable high-resolution 3D imaging, offering potential improvements in diagnostic accuracy, efficiency, and objectivity.^35,36^

 Accurate evaluation of gingival surface characteristics is crucial for periodontal diagnosis and monitoring. Conventional approaches, such as visual inspection, probing, and intraoral photography, are limited to site-specific information; while probing records pocket depth and bleeding, it does not capture overall gingival morphology or swelling. In contrast, intraoral scanning (IOS) enables full-arch visualization of gingival contours, color, and texture, supporting comprehensive and longitudinal assessment. Previous studies using digital impressions (e.g., Lava COS) have shown short-term improvements in gingival health following professional prophylaxis, evidenced by reductions in modified gingival index (MGI), bleeding index (BI), and gingival contour volume, with effects peaking at six weeks and diminishing by 12 weeks, highlighting the transient benefit without sustained plaque control. Improvements at non-treated sites were likely due to enhanced oral hygiene practices. Although clinical indices showed greater improvements at six weeks compared to four weeks, this was not consistently reflected in contour volume measurements, suggesting a true discrepancy that may affect correlations. Nonetheless, reductions in contour volume generally paralleled clinical improvement, indicating its value as an objective, quantitative parameter for gingivitis assessment, best used as a complement to conventional clinical indices.^13^

 Accurate superimposition of serial digital records is critical for longitudinal assessment of periodontal and orthodontic outcomes. IOS-based mandibular digital model registration demonstrates high intra- and inter-rater reliability, with accuracy comparable to voxel-based CBCT. Similar measurements between anterior CBCT and digital models support the use of baseline CBCT followed by serial IOS scans to monitor hard and soft tissues while reducing radiation exposure. The mucogingival junction serves as a stable reference for reproducible superimposition, though accuracy may decrease in cases of extensive tooth movement or advanced periodontal destruction.^14^

 Assessing soft tissue accuracy is challenging because there is no true in vivo reference standard. Conventional impressions are prone to tissue compression, deformation, and material instability, particularly in marginal and papillary regions, reducing precision. In contrast, intraoral scanning (IOS) eliminates contact-related distortion and offers comparable or superior accuracy for short-span 3D gingival assessment, especially in the anterior maxilla. However, accuracy may decline in distal and edentulous areas due to stitching errors and limited reference points.^15^ Digital accuracy, therefore, requires evaluation of both trueness, measured by mean positive and negative deviations, and precision, assessed using percentile-based deviation metrics that reflect overall surface discrepancies.^37‒42^

 In the present review, measurement variability is further influenced by differences in software algorithms, segmentation approaches, and pixel resolution, which remain major sources of inconsistency across studies. Soft tissue reflectance and color variability also affect the accurate detection of inflammation. Conventional periodontal probing exhibits variability of approximately ± 1 mm, whereas IOS-based measurements are often reported within the 80‒150 µm range. Without contextualizing these thresholds, claims of “accuracy” should be interpreted cautiously.

 Several studies have validated IOS for gingival health assessment. Trios 3Shape IOS demonstrated high accuracy compared with conventional MGI scoring, with excellent intra- and inter-examiner agreement and no clinically relevant deviations exceeding one MGI point. Minor overestimation of inflammation likely reflected the subjectivity of visual assessment.^16^ Binary color and texture evaluations derived from IOS matched clinical findings perfectly, highlighting the scanner’s ability to discriminate soft tissue characteristics. With broader adoption of IOS, clinicians can obtain non-invasive, full-mouth 3D records that facilitate consistent longitudinal monitoring, reduce examiner dependence, and support future algorithm-based classification of gingivitis, while also enhancing patient understanding and motivation.^16^

 Digital plaque assessment has significantly benefited from intraoral scanner (IOS) technology, demonstrating substantial to near-perfect reliability when compared with 2D intraoral photographs and conventional clinical indices. The Rustogi Modified Navy Plaque Index (RMNPI) has shown high sensitivity in detecting even minimal plaque deposits. Both IOS and 2D imaging tend to yield slightly higher plaque scores than direct clinical examination, likely due to enhanced magnification, illumination, and access. Agreement with clinical findings is excellent for 2D images and good for 3D IOS images, although reduced accuracy is noted on lingual and mandibular posterior surfaces due to scanner and software constraints.^17,43^

 However, the findings from the present meta-analysis should be interpreted with caution. Although a numerical difference in plaque scores was observed, its clinical relevance remains unclear because a well-defined minimum clinically important difference (MCID) for plaque indices has not been established. Therefore, the observed difference cannot be directly translated into meaningful clinical benefit. While no standardized MCID exists for plaque indices such as the Rustogi Modified Navy Plaque Index or Quigley-Hein Plaque Index used in the included studies, limited literature has explored patient-reported or clinically meaningful thresholds for plaque reduction in gingivitis trials (e.g., 20‒30% relative change in plaque scores correlating with perceptible gingival improvements). However, these remain unvalidated for diagnostic accuracy comparisons.

 Compared with intraoral camera images, which are limited by incomplete surface capture, proximal distortion, and uneven lighting, IOS provides more comprehensive and uniformly illuminated surface visualization. This often results in systematically higher plaque percentages, as confirmed by Bland-Altman analyses. Measurement variability may arise from scan orientation, cropping, and plaque-disclosing dye retention, particularly on mandibular oral surfaces, and repeated staining may increase post-brushing scores. Despite these limitations, plaque distribution patterns observed with IOS closely align with established clinical expectations, supporting its validity as a reliable tool for both plaque quantification and patient education.^18^

 Beyond assessment, IOS data have been used to develop innovative patient-centered tools. A fully automated 3D gingival recession simulation system enables visualization of patient-specific recession scenarios based on selected attachment loss parameters. Using automated gingival-margin detection and advanced geometric algorithms, the system overcomes limitations of earlier semi-automated approaches and incorporates periodontal risk classification. Such tools have significant potential to improve patient motivation, compliance, and understanding of disease progression.^19^

 Clinical studies further demonstrate that IOS-guided interventions can enhance oral hygiene outcomes. Tailored oral hygiene programs incorporating IOS images of disclosed plaque, sodium bicarbonate toothpaste, and regular reminders produced significantly greater reductions in bleeding on probing (BOP) and plaque compared with standard advice. Improvements were most pronounced on lingual and palatal surfaces, which initially exhibited the poorest hygiene. IOS visualization allowed patients to identify missed areas and adjust brushing techniques accordingly. Although BOP reductions exceeded plaque reductions, possibly reflecting a Hawthorne effect, sustained plaque control was associated with improved gingival health. Importantly, IOS images were suitable for reliable remote plaque scoring, reducing inter-clinician variability.^20^

 Digital workflows have also proven valuable in aesthetic and surgical planning. Digital and analog methods showed comparable accuracy in locating the gingival zenith, with digital workflows reducing operator-related error. Findings confirmed contralateral symmetry and established patterns in zenith position, reinforcing their relevance in smile design. Similarly, integrating CBCT and IOS enables accurate, non-invasive measurement of palatal mucosal thickness, which is critical for soft tissue grafting in aesthetic implant zones. Digital measurements demonstrated high reliability and close agreement with reference standards, supporting their clinical applicability.^21^

 Assessment of periodontal phenotype has traditionally relied on invasive transmucosal probing. Digital superimposition techniques combining DICOM and STL data have shown moderate to very high correlations with direct measurements of gingival thickness, providing a reliable and reproducible non-invasive alternative for measuring palatal mucosal thickness in the maxillary anterior region.^24^ By measuring gingival and bone thickness at identical reference levels, these methods ensure valid comparisons and broaden applicability across all tooth types. Although digital assessments require greater resources and expertise, their accuracy depends primarily on data quality and standardized protocols.^25^

 Advances in automation and artificial intelligence further address limitations of conventional diagnostics. Fully automated systems integrating IOS-derived soft tissue morphology with CBCT-based bone data can directly measure gingival–bone distance in 3D, providing continuous circumferential assessments that outperform traditional six-site probing. Such systems demonstrate high segmentation accuracy and minimal measurement error, offering a more complete and objective representation of periodontal status.^26^

 Deep-learning approaches using IOS data have also shown promise for assessing gingival inflammation. Models such as GC-U-Net outperform conventional architectures in identifying gingival swelling and demonstrate strong correlations with clinical bleeding indices. While these systems are more effective for surface inflammation than deep periodontal pathology, they serve as valuable adjuncts for targeted plaque control, patient education, and risk visualization. Future improvements through multimodal integration and larger datasets are likely to enhance robustness and clinical relevance.^27^

 Finally, IOS has clear advantages over traditional plaque indices and photographic documentation. While indices such as the Plaque Control Record are widely used, they are limited by operator variability and lack permanent records. IOS offers detailed, reproducible digital documentation with minimal discomfort and efficient single-operator use. Although scanning posterior regions remains challenging due to anatomical constraints and the size of the scanner tip, ongoing hardware and software improvements are expected to mitigate these limitations.^28^

 In summary, evidence suggests acceptable accuracy, reliability, and clinical utility of IOS-based digital models for periodontal assessment, plaque detection, soft tissue measurement, and patient education. While digital methods should complement rather than replace conventional clinical indices, their integration into routine practice has the potential to improve diagnostic objectivity, reduce examiner variability, enhance patient engagement, and support longitudinal monitoring. Continued technological refinement, standardized protocols, and larger clinical studies will further define their role in comprehensive periodontal care. Generalizability remains limited, as most studies utilized specific scanner systems (primarily TRIOS and iTero) and were conducted in populations with mild gingival inflammation.

 Limitations of the present review include that soft tissue is inherently dynamic and susceptible to saliva, inflammation, tissue mobility, and patient movement, resulting in surface deviations, particularly in unattached mucosa and posterior regions. Scanner size, limited access, and line-of-sight constraints reduce accuracy on lingual and distal surfaces, especially in the mandible. In edentulous areas, the lack of stable anatomical landmarks compromises scan stitching and superimposition. Measurement accuracy is further influenced by scanning protocols, operator experience, software algorithms, and model reconstruction, which can introduce systematic errors. The absence of a true in vivo reference standard limits the definitive validation of IOS soft tissue accuracy. Furthermore, differences in reference standards used across studies (clinical probing, photographic analysis, or CBCT-based measurements) limited the ability to perform pooled analysis for several outcomes.

 Additionally, the conventional clinical reference standards used across included studies, such as periodontal probing and clinical indices, are themselves subject to inherent variability due to differences in probe type, probing force, angulation, and examiner-dependent factors; this represents a recognized contributor to diagnostic heterogeneity in comparative IOS assessments. Soft tissue color variability and reflectance differences also affect the accuracy of inflammation assessment using IOS-based imaging. Many studies are constrained by small sample sizes, short follow-up periods, and single-center or single-examiner designs. In addition, most included studies were observational and exhibited moderate-to-high risk of bias, limiting the strength of causal inference. Variability in outcome definitions and the absence of standardized measurement units across studies further restrict the direct comparability of findings. Additionally, digital and AI-based systems require substantial investment in equipment, software, training, and time. While AI shows promise, performance may decline in low-contrast images, complex anatomical conditions, or advanced periodontal defects, and it cannot yet replace clinical probing for deep periodontal assessment. Given the heterogeneity in study designs and outcome measures, meta-analysis was limited to plaque-related outcomes only and should be interpreted with caution.

 Future research should prioritize large, multicenter longitudinal studies to validate IOS-based periodontal assessment across diverse populations and disease severities. The development of standardized scanning protocols, reference landmarks, and analytical methods is essential to improve reproducibility. Standardization efforts should specifically address plaque segmentation thresholds, gingival volume quantification algorithms, and consistent landmark definitions for recession measurement to enable cross-study comparability. Harmonization of digital file formats (e.g., STL, OBJ, and PLY) is also necessary to ensure interoperability across different software platforms. Technological advancements such as smaller scan heads, faster acquisition, higher resolution, and improved soft tissue segmentation are expected to mitigate current limitations. Integrating multimodal data from IOS, CBCT, clinical indices, and biological markers may enhance diagnostic accuracy and risk prediction. Continued refinement of AI-driven tools should focus on robust performance in complex clinical scenarios, improved detection of deep periodontal pathology, and real-time decision support. Evaluation of cost-effectiveness, workflow efficiency, and patient-reported outcomes will be critical for broader clinical adoption. Future studies should also evaluate practical workflow considerations, including scanning time, data processing requirements, software costs, and long-term data storage implications, which may influence real-world clinical adoption.

## Conclusion

 IOS digital models represent a major advancement in periodontal diagnosis, monitoring, and patient management. Evidence supports their accuracy and reproducibility in assessing gingival morphology, plaque distribution, periodontal phenotype, and soft tissue dimensions, with seamless integration with CBCT for comprehensive analysis. Compared with conventional methods, digital workflows reduce subjectivity and examiner dependence while enabling non-invasive, full-arch, and longitudinal evaluation. Although clinical probing remains indispensable, IOS-derived metrics such as gingival volume, digital plaque quantification, and gingiva‒bone distance provide valuable complementary information. Enhanced visualization and emerging AI applications improve patient education and motivation, supporting behavioral change. IOS should therefore complement rather than replace conventional clinical probing, and current evidence, particularly for recession and defect detection, should be interpreted cautiously, given limited and heterogeneous quantitative data.

## Competing Interests

 The authors declare that they have no competing interests regarding the authorship and/or publications of this paper.

## Data Availability

 The data that support the findings of this study are available from the corresponding author upon reasonable request.

## Ethical Approval

 Not applicable.
